# Carotid intima-media thickness is not related with clinical outcomes in young hypertensives

**DOI:** 10.1186/s40885-015-0021-x

**Published:** 2015-07-22

**Authors:** Jin-Sok Yu, Yun-Seok Choi, Ju-Youn Kim, Ji-Hee Kim, Woo-Baek Chung, Chul-Soo Park, Yong-Seog Oh, Ho-Joong Youn, Wook-Sung Chung, Man-Young Lee

**Affiliations:** Department of Internal Medicine, The Catholic University of Korea, Yuksamro 10, Seoul, 150-713 South Korea

**Keywords:** Carotid intima-media thickness, Hypertension

## Abstract

**Introduction:**

Careful observations of long- and short-term outcomes associated with carotid intima-media thickness (IMT) are relatively limited.

**Methods:**

A total of 2,972 patients (male:female = 1,960:1,012; mean age = 62 ± 12 years) who underwent carotid IMT measurements from September 2003 to March 2009 were divided into four groups. Group I (*n* = 271; mean age, 42 ± 7.8 years) included normotensive younger subjects (males, <45 years and females <55 years), group II (*n* = 992; mean age, 63 ± 9 years) included normotensive elderly subjects, group III (*n* = 177; mean age, 46 ± 7.8 years) was hypertensive younger subjects, and group IV (*n* = 1,532; mean age, 63 ± 10.2 years) was hypertensive elderly subjects. We analyzed the clinical and cardiovascular events in the younger hypertensive subjects based on IMT measurements.

**Results:**

The baseline characteristics of the subjects showed that carotid IMT increased in the elderly subjects and in patients with hypertension. Poor clinical outcomes, such as all-cause death and major adverse cardiac events, were related with age, not with hypertension. Among the conventional risk factors, age and the highest quartile level of right maximum carotid IMT were related with major adverse events (young: odds ratio [OR], 0.47; 95% confidence interval [CI], 0.25 to 0.9 vs. OR, 1.73; 95% CI, 1.20 to 2.49). The patients in the highest quartile of carotid IMT had worse survival outcomes than those with the lowest IMT (*p* = 0.03).

**Discussion:**

Subjects with hypertension had increased carotid IMT levels. Controlling hypertension and carefully evaluating carotid IMT are important to prevent cardiovascular events even in younger subjects with hypertension.

## Introduction

Measurements of carotid intima-media thickness (IMT) have been used as a surrogate screening test for cardio-cerebrovascular disease [1,2]. However, careful observations of long- and short-term outcomes associated with IMT are relatively limited. Thus, it is quite important to define the clinical significance of IMT as a screening test in a large cohort study. Moreover, this screening test is imperative, considering the socio-economic significance of cardiovascular events in relatively young subjects with hypertension. IMT measurements reflect changes that occur early in atherosclerosis rather than those in advanced disease. Most young subjects with hypertension show early vascular changes because of short exposure, indicating that IMT may be useful to detect these early vascular changes that may be associated with long-term outcome.

Measuring IMT using ultrasonography is simple and safe, uses no radiation, and is an inexpensive test to indirectly examine the presence of coronary atherosclerosis [3,4]. Noninvasive high-frequency ultrasonography produces valid estimates of total wall thickness and MT in the carotid arteries [5]. Associations between cardiovascular risk factors and IMT have been established across a wide age range, including children and adults [6-9]. Changes occurring in the thickness of the intima and media layers are distinct from each other with advancing age and through the development of atherosclerosis [10,11]. The aim of this study was to determine the clinical usefulness of measuring carotid IMT in relatively young patients with hypertension.

## Methods

### Study population

A total of 2,972 patients (1,960 males and 1,012 females; mean age, 62 ± 12 years), who underwent IMT measurements at St. Mary’s Hospital from September 2003 to March 2009, were enrolled in this study. All patients underwent B-mode carotid artery ultrasound and had hypertension regardless of medication use.

Group I (*n* = 271; mean age, 42 ± 7.8 years) included younger subjects (males <45 years and females <55 years) without hypertension, group II (*n* = 992; mean age, 63 ± 9 years) was elderly subjects without hypertension, group III (*n* = 177; mean age, 46 ± 6.2 years) was younger subjects with hypertension, and group IV (*n* = 1,532; mean age, 63 ± 10.2 years) was elderly subjects with hypertension. We also compared cardiovascular risk factors, such as smoking, diabetes mellitus, and dyslipidemia (total cholesterol ≥ 220 mg/dL, triglycerides ≥ 150 mg/dL) among the groups. Hypertension was defined as systolic blood pressure ≥140 mmHg and diastolic blood pressure ≥90 mmHg on an office measurement or a current prescription for hypertensive medication. We analyzed long-term outcomes and cardiovascular events in the younger subjects with hypertension according to IMT level. We contacted patients who could not attend the follow-ups by telephone. Death was defined as all-cause death. Major adverse cerebro-cardiac events included death, stroke (including transient ischemic attack [TIA]), and non-fatal myocardial infarction. We also evaluated the predictive value of IMT on survival risk. The ethics committee of the institutional board (The Catholic University Institutional Review Board) approved the use of clinical data for this study, and all patients provided written informed consent (KC10RISI0585).

### IMT measurements

The carotid arteries were evaluated using high-resolution B-mode ultrasound with a 15-MHz linear array transducer (HP Sonos-5500; Philips, Bothell, WA, USA). Carotid arterial scanning was performed by two certified sonographers who were blinded to all clinical information. The patients were placed in the supine position with slight hyperextension and rotation of the neck to the contralateral side, and the IMT measurements were obtained through 10-mm segments across the far wall of the right common carotid artery at the point most proximal to the carotid bifurcation. We selected a region of interest (ROI) usually within the proximal 20 mm and distal 15 mm from the carotid bulb that was free of plaque. Maximum IMT was defined as the maximum IMT value in the ROI on both sides of the carotid artery.

Transducer frequency was 15 MHz during the entire analysis with axial resolution of 0.2 mm. Measurements were acquired at the end-diastolic phase (defined as the electrocardiogram R wave) because systolic expansion of the lumen causes IMT thinning [11,12]. IMT was defined as the distance between the luminal border of the intima and the outer border of the media. IMT was assessed by manually measuring thickness on all plaque-free lesions. Calipers were placed six times for individual measurements, and the mean value was calculated.

IMT values were analyzed using inter-quartile range. We also compared survival benefit between the highest and lowest quartiles. The left IMT was not included.

### Blood pressure measurements

Blood pressure was measured with a mercury sphygmomanometer after subjects had rested comfortably for 5 min in a sitting position. The mean of three measurements was used for analyses. The blood pressure measurements were performed immediately before and after the IMT examination. Systemic hypertension was defined as systolic pressure ≥140 mmHg and/or diastolic pressure ≥90 mmHg, based on more than three measurements or current use of an antihypertensive drug.

### Statistical analysis

Continuous variables are presented as mean ± standard deviation, and categorical variables are presented as absolute values and relative frequencies (%). The cardiovascular risk factor data were compared using the independent Student *t*-test for continuous variables. Categorical variables were compared using the chi-square test. Cardiovascular risk factors and IMT were assessed with Pearson’s correlation coefficient analysis. A multivariate linear regression analysis was performed to determine the independent risk factors for IMT. All reported *p* values were two-sided, and *p* values <0.05 were considered significant. Intra-class correlation coefficient (ICC) analysis is a statistical method used to measure intra- and inter-observer reliability. Reliability was assessed by replicating measurements for 50 patients. The ICC assesses the consistency of multiple measurements of the same quantity. All statistical analyses were conducted using SAS ver. 9.1 statistical software (SAS Institute Inc., Cary, NC, USA).

## Results

### Clinical characteristics

The baseline characteristics of the 2,972 patients are shown in Table 1. Mean age of the study subjects was 42 ± 7.8 years, and 53.5% were men. All patients underwent carotid measurements at both carotid arteries. Younger patients with hypertension developed stroke at a rate of 1.7%, whereas that in the elderly subjects was 3.4% (*p* > 0.05). IMT values were significantly higher in elderly subjects and those with hypertension (Table 1). IMT values in the elderly subjects with normotension were larger than those in younger subjects with hypertension.

**Table 1:** **Baseline characteristics**

**Characteristic**	**HTN (−)**		**HTN (+)**	
	**Younger (n = 271)**	**Elderly (n = 992)**	**Younger (n = 177)**	**Elderly (=1,532)**
Age (year)	42 ± 7.8	63 ± 9	46 ± 7.8	63 ± 10.2
Gender (male)	155 (56.6)	689 (68.3)	63 (35.6)	873 (56.0)
Non-insulin dependent diabetes mellitus	30 (11)	243 (24.1)	51 (30.5)	550 (37.6)
Smoking	61 (29.7)	202 (25.0)	32 (23.4)	281 (21.3)
Death	1 (0.4)	30 (3.0)	0	52 (3.4)
Stroke	1 (0.4)	31 (3.1)	3 (1.7)	51 (3.4)
Major adverse cerebrovascular events	9 (3.6)	77 (7.7)	4 (2.2)	111 (7.4)
Total cholesterol (mg/dL)	175 ± 42	169 ± 41	179 ± 43	169 ± 47
High-density lipoprotein (mg/dL)	45 ± 11	44 ± 11	46 ± 13	43 ± 11
Triglycerides (mg/dL)	130 ± 90	135 ± 91	165 ± 122	146 ± 98
Creatinine (mg/dL)	0.97 ± 0.49	1.07 ± 0.36	1.06 ± 0.90	1.19 ± 0.91
Right maxIMT (mm)	0.77 ± 0.19	0.98 ± 0.25	0.85 ± 0.22	1.03 ± 0.29
Right meanIMT (mm)	0.66 ± 0.15	0.84 ± 0.19	0.73 ± 0.16	0.87 ± 0.20
Left maxIMT (mm)	0.77 ± 0.20	1.00 ± 0.26	0.84 ± 0.21	1.03 ± 0.26
Left meanIMT (mm)	0.68 ± 0.17	0.87 ± 0.21	0.75 ± 0.19	0.90 ± 0.22

Values are presented as mean ± standard deviation or number (%). maxIMT, maximal intima-media thickness.

### Prediction of major adverse cerebro-cardiac events

We divided the carotid IMT values according to quartiles to compare clinical outcomes (Table 2). Subjects in the highest quartile for right maximum IMT values or who were younger were significantly associated with predicting cerebro-cardiovascular events. We minimized the interaction by comparing statistically between the groups. However, subjects in the highest quartile for the left maximum IMT value were statistically significant (Table 3).

**Table 2:** **Inter-quartile range of IMT**

**Variable**	**Range**	**25th**	**50th (median)**	**75th**
No. of patients (right)		630	1,721	621
Right maxIMT (mm)	0.32–3.22	0.79	0.95	1.11
Right meanIMT (mm)	0.34–2.25	0.62	0.69	1.09
No. of patients (left)		215	1,448	203
Left maxIMT (mm)	0.42–2.07	0.66	0.78	1.30
Left meanIMT (mm)	0.26–1.67	0.64	0.83	1.03

IMT, intima-media thickness; maxIMT, maximal IMT.

**Table 3:** **Prediction of major adverse cerebro-cardiac event**

**Variable**	**Hazard ratio**	**95% confidence interval**	**p value**
Younger age	0.47	0.25–0.90	0.02
Hypertension	3.25	0.77–13.66	0.10
Non-insulin dependent diabetes mellitus	1.57	0.31–7.92	0.58
Smoking	1.59	0.12–2.98	0.97
Right maxIMT highest QR	1.73	1.20–2.49	0.03
Left maxIMT highest QR	1.02	0.92–1.41	0.11

maxIMT, maximal intima-media thickness; QR, quartile range.

### Survival comparison according to carotid IMT level

**Figure 1:**
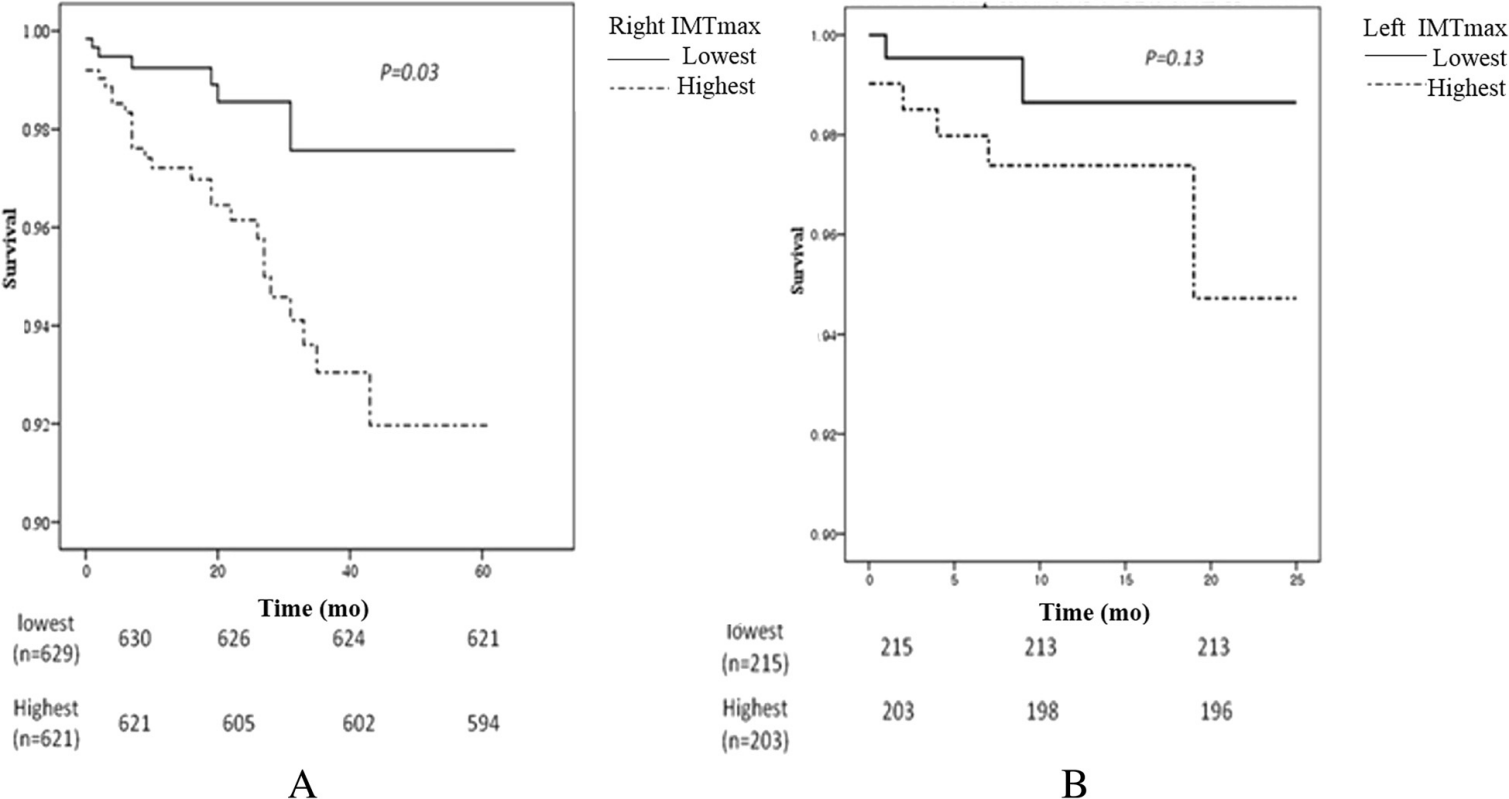
Comparison of survival benefit according to the level of carotid IMT. **(A)** The highest quartile value of right maximal IMT group showed the survival benefit compared to lowest group. **(B)** Left maximal IMT did not reach the statistical significance. IMT, intima-media thickness.

The carotid IMT values were divided by quartiles. Subjects in the right maximum highest IMT quartile had a significantly poorer survival rate than those in the lowest quartile (*p* = 0.03). However, the highest left carotid IMT group did not show significance. At the beginning of our cohort, we only measured the right carotid IMT. So the follow-up duration was different. The median duration of follow-up was 43 months (range, 6 to 62 months) in the patients with maximum right IMT levels (Figure 1).

### Younger subjects with hypertension

**Figure 2:**
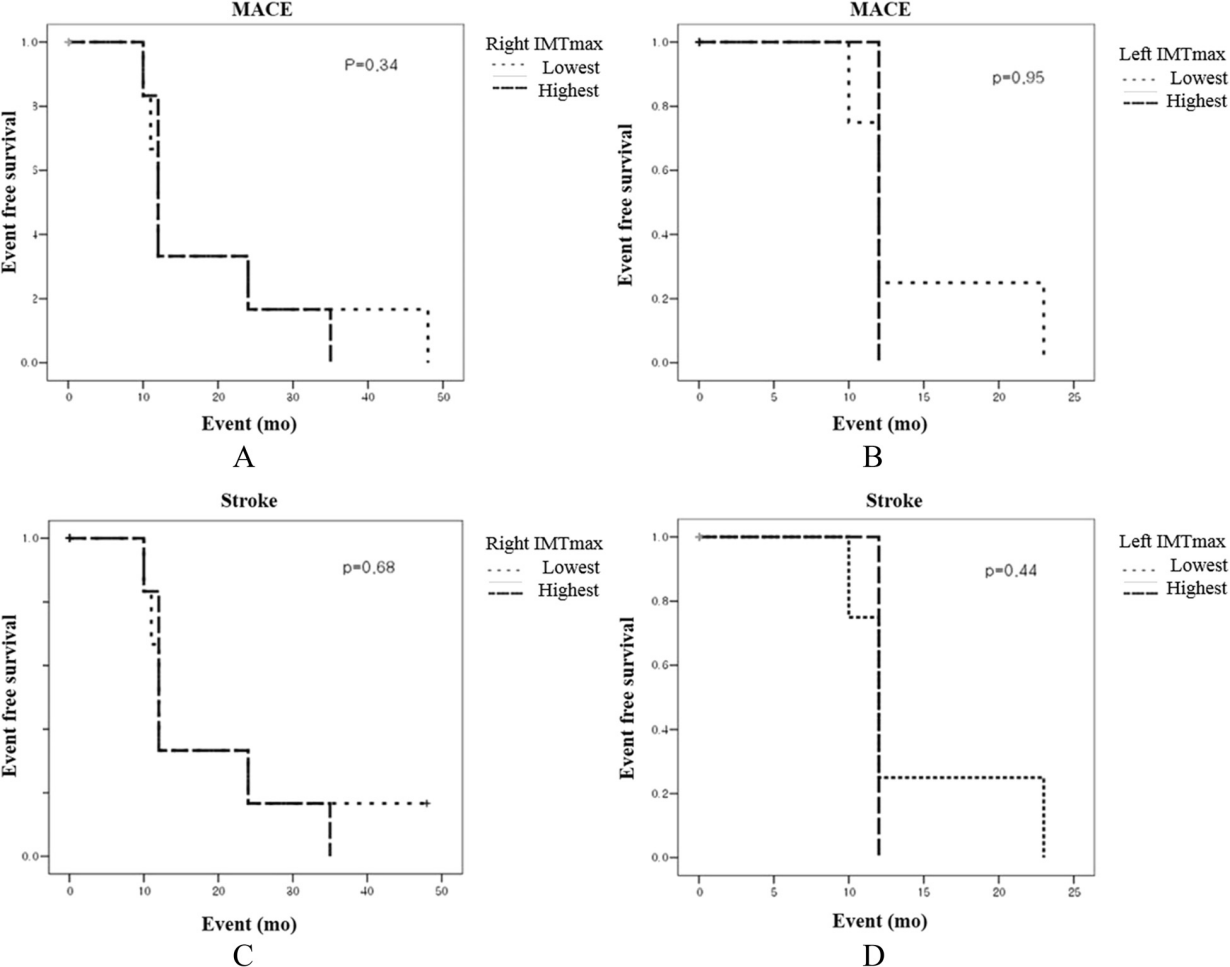
Comparison of survival benefit according to the quartile range of carotid IMT. There were no significant differences in terms of **(A** and **B)** MACE or **(C** and **D)** stroke between the highest and lowest level of maximal IMT. MACE, major adverse cerebrovascular events; IMT, intima-media thickness.

Mean disease duration in the 177 younger patients with hypertension was 7 ± 9 years. Most of these patients (93%) were taking antihypertensive medication. The IMT values of these patients are shown in Table 4. Carotid IMT values in subjects with hypertension were significantly higher compared to those of subjects with normotension, regardless of age. No significant differences in clinical outcomes from major adverse cardiac events, including death, myocardial infarction, and stroke, were observed between the highest and lowest IMT values based on the inter-quartile range in the younger subjects with hypertension (Figure 2).

**Table 4:** **Carotid IMT in young hypertensives**

**Variable**	**HTN (−) (n = 271)**	**HTN (+) (n = 177)**	**p value**
Right maxIMT (mm)	0.77 ± 0.19	0.85 ± 0.22	0.012
Right meanIMT (mm)	0.66 ± 0.15	0.73 ± 0.16	0.018
Left maxIMT (mm)	0.77 ± 0.20	0.84 ± 0.21	0.020
Left meanIMT (mm)	0.68 ± 0.17	0.75 ± 0.19	0.031

HTN, hypertension; maxIMT, maximal intima-media thickness.

### Inter- and intra-observer reliability of the measurements

Intra- and inter-observer reliability for the IMT measurements, as measured by the ICC, was 0.965 and 0.900, respectively.

## Discussion

The value of carotid IMT differs substantially according to epidemiological variables, such as race, age, and the presence of disease. Thus, normal reference values must be determined in large Korean cohorts to predict cerebro-cardiac events. This was a large-scale study that included a variety of atherosclerotic risk factors.

We divided the patients into four groups according to age and hypertension. Clinical events, such as stroke and major adverse cerebrovascular events (MACE), were associated with old age, not hypertension, which may have been due to the relatively short follow-up duration and small population. However, hypertension clearly affected IMT thickness. We found that MACE was significantly associated with age and right maximum IMT thickness when we compared the carotid IMT values of younger subjects with hypertension with those of elderly subjects with normotension. But in the young hypertensives, most cardiac death did not occur during the follow-up period and most MACE were related with stroke including TIA. This result indicates that the highest right maximum IMT was predictive of MACE. IMT measurements are widely used to detect cardiovascular disease, as they are very easy to obtain, and the test is noninvasive. Thus, IMT has an important clinical role in preventing atherosclerosis by detecting patients with high IMT values. The left highest IMT quartile value was not associated with long-term outcome. Many studies have suggested why left-side IMT thickness has a poorer relationship with clinical outcomes than that of the right; however, this has not been fully elucidated [12]. We also found that the highest value of the right maximum IMT predicted a poorer clinical outcome compared to that of the lowest value, suggesting that it is important to prevent progression of IMT thickening. Actually, thickening of carotid IMT tends to progress very slowly. Thus, it was difficult to detect the clinical and survival benefits through a shorter follow-up. However, we did find a relationship between highest IMT quartile.

IMT is strongly related to cardiovascular risk factors and early stages of vascular atherosclerosis in young adults [13]. Increased IMT thickness is associated with hypertension due to medial hypertrophy or fibromuscular hyperplasia associated with aging [14,15]. In the present study, age was closely correlated with IMT. Although we intended to show the relationship between carotid IMT and outcomes in various subject groups of hypertensives according to age, we cannot conclude that younger subjects with hypertension had poorer outcomes compared to those of elderly subjects with or without hypertension. We can only say that younger subjects with hypertension showed significantly thickened carotid IMT even though they had relatively short exposure to hypertension and had a paucity of cardiovascular risk factors. This result indicates that controlling hypertension and managing risk factors thoroughly as early as possible leads to a better clinical outcome and that it should be started as soon as possible.

Some limitations of our study deserve mention. First, we measured IMT in the far walls of both common carotid arteries because measuring IMT in the common carotid as well as the carotid bulb segments is more useful for detecting early stages of atherosclerosis and hypertensive changes in young adults [16]. Second, measuring IMT using manual tracing and ultrasound imaging is operator-dependent. We used the ICC to evaluate intra- and inter-observer reliability. Third, the number of young patients may have been insufficient to detect small differences in long term outcomes. Moreover, we could not elucidate the clinical meaning of hypertension in pre-specified younger patients prospectively. Fourth, we could not elucidate the influences of other risk factors that could be related to long-term outcome. Finally, the definition of younger was determined arbitrarily.

## Conclusions

In conclusion, our results show that carotid IMT measurements using a high-frequency transducer are predictive for cardiovascular events. Carotid IMT increased even in younger subjects with hypertension and may be related with poor clinical outcome.
